# Liu Sung-nien (1174–1224), Sung Dynasty. Lohan (1207)

**DOI:** 10.3201/eid1002.AC1002

**Published:** 2004-02

**Authors:** Polyxeni Potter

**Affiliations:** * Centers for Disease Control and Prevention, Atlanta, Georgia, USA

**Figure:**
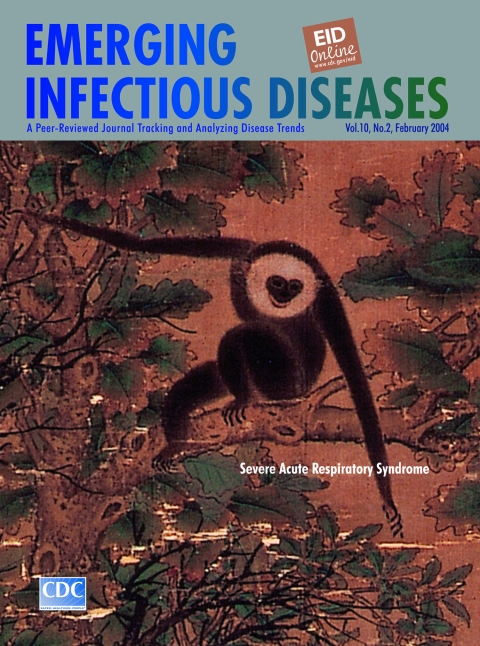
Liu Sung-nien (1174–1224), Sung Dynasty. Lohan (1207) National Palace Museum, Taiwan, Republic of China. Hanging scroll, ink and colors on silk (117 cm x 55.8 cm)

**Table:** 

Liu Sung-nien was a court painter from Ch'ien-t'ang (modern Hangchow). He painted figures and landscapes and was honored by Emperor Ning-tsung during the Sung Dynasty (960–1279), a prosperous and culturally rich period of Chinese history. The arts (silk tapestries, embroideries, calligraphy, lacquer, porcelain, pottery) explored the complexities of the physical and spiritual world, and painting reached full maturity in its reverence for nature and keen observation of life (*1*,*2*).

The Sung Dynasty embraced education and erudition and nurtured many cultural luminaries, who pondered the universe but walked among the people and had a down-to-earth accessible style. The lohans, Buddhist ascetics whose meditative personas inspired the pursuit of compassion and enlightenment, became a popular subject in paintings. Three of Liu's lohan works are in the National Palace Museum in Taiwan. The hanging scroll on this month's cover of Emerging Infectious Diseases is one of them.

In its entirety, the painting reflects harmonious human interaction with nature. The lightness of the scene, achieved through subtle brushstrokes and fluid, diaphanous earth hues, is punctuated by the playful presence of animals in an intimate ensemble, humans in the center, deer in the foreground, gibbons frolicking in the foliage above. The aged branches form a wrinkled halo around the lohan, who seems lost in thought. The cozy scene, intricately structured within an ancient pomegranate tree, embraces the sage and his acolyte in a warm give-and-take with the gibbons, while back-to-back, the deer gaze upward, one at the lohan, the other at the gibbons.

Elaborate detail is present throughout the scroll, including the upper part (cover selection) that crowns the pleasant scene. Yet, the creatures in the branches are abstract and stylized. The heart-shaped faces, characteristically long, tapering arms, and deep, humanlike eyes depict the essence but omit the details. Unlike the lohan, whose age and calling are dexterously outlined in his prominent visage, they are representational. On the periphery yet not to be ignored, they perform their acrobatic game with grace and confidence. They pick from the ancient tree and toss into the scene a pomegranate, thought by many to be the biblical fruit of knowledge (*3*).

Primates are common inhabitants of art scenes and feature frequently in Chinese literature. According to one legend, they, along with other animals, were once invited to a celebration held by the Jade Emperor of Heaven. Among the first 12 animals to arrive, monkeys were named part of the zodiac and were assigned a year on the Chinese solar/lunar calendar (*4*).

Liu's colorful scroll of nature nestled in the ancient pomegranate tree is a tempting metaphor for our times. The tart exotic fruit, its ageless perseverance within the leathery skin and its allusion to knowledge within the neatly membraned clusters of scarlet seeds, conveys optimism. The scene's moment of hilarity and harmony sends a message of community, where the answers to complex questions are collective and may well come from entirely unexpected places. While the lohan turns inward to think, the gibbon, like deus ex machina, passes the clue to the next of kin, who in turn will toss it over to those who have eyes to see.

Knowledge, a communal effort laboriously assembled piece by piece, relies on swift and purposeful give and take. Non-human primates more than once have held valuable clues to human puzzles, from AIDS to hepatitis. Sometimes the vehicle, but more often the oracle of zoonotic scourges, they have shared with us generously. In this the Chinese Year of the Monkey, the long arm of the gibbon may yet reach across the seas with seeds of knowledge for the global health community deciphering the puzzle of SARS.
